# An RBF neural network based on improved black widow optimization algorithm for classification and regression problems

**DOI:** 10.3389/fninf.2022.1103295

**Published:** 2023-01-10

**Authors:** Hui Liu, Guo Zhou, Yongquan Zhou, Huajuan Huang, Xiuxi Wei

**Affiliations:** ^1^College of Artificial Intelligence, Guangxi University for Nationalities, Nanning, China; ^2^Guangxi Key Laboratories of Hybrid Computation and IC Design Analysis, Nanning, China; ^3^Department of Science and Technology Teaching, China University of Political Science and Law, Beijing, China; ^4^Xiangsihu College of Guangxi University for Nationalities, Nanning, China

**Keywords:** improved black widow optimization algorithm, radial basis function neural networks, classification and regression, power load prediction, metaheuristic

## Abstract

**Introduction:**

Regression and classification are two of the most fundamental and significant areas of machine learning.

**Methods:**

In this paper, a radial basis function neural network (RBFNN) based on an improved black widow optimization algorithm (IBWO) has been developed, which is called the IBWO-RBF model. In order to enhance the generalization ability of the IBWO-RBF neural network, the algorithm is designed with nonlinear time-varying inertia weight.

**Discussion:**

Several classification and regression problems are utilized to verify the performance of the IBWO-RBF model. In the first stage, the proposed model is applied to UCI dataset classification, nonlinear function approximation, and nonlinear system identification; in the second stage, the model solves the practical problem of power load prediction.

**Results:**

Compared with other existing models, the experiments show that the proposed IBWO-RBF model achieves both accuracy and parsimony in various classification and regression problems.

## 1. Introduction

There are many existing solutions to classification problems. For example, classifiers based on fuzzy logic, advanced and state-of-the-art (SOTA) deep learning, and artificial intelligence (AI) methods. Fuzzy neural classifier is developed for disease prediction in Kumar et al. (2018). Versaci et al. (2022) proposed a fuzzy similarity-based approach for classifying carbon fiber-reinforced polymer plate defects. Belaout et al. (2018), a multiclass adaptive neuro-fuzzy classifier (MC-NFC) was developed for fault detection and classification in photovoltaic array. Semi-Active Convolutional Neural Networks are developed for Hyperspectral Image Classification in Yao et al. (2022). Zhang H. et al. (2022) proposed the convolution neural network method with the attention mechanism for classification of hyperspectral and light detection and ranging (LiDAR) data. Hong et al. (2020) proposed multimodal generative adversarial networks for image segmentation. The classification method adopted in this paper is a hybrid method of black widow optimization (BWO) algorithm and radial basis function neural network (RBFNN), and it is used in several regression problems to verify the performance.

Radial basis function neural network is a kind of artificial neural network (ANN) with activation functions based on Gaussian kernels, which has the advantages of simple structure, easy parameter adjustment and strong generalization ability (Broomhead and Lowe, 1988). The key to improving the performance of ANN is to search for the optimal parameters. Methods for training ANN parameters include gradient-based algorithms and metaheuristic algorithms, the latter of which are more likely to escape from the local optimum (Wu et al., 2016). There has been a great deal of research on combining metaheuristic algorithms with ANNs for a variety of classification and regression problems. For example, particle swarm optimization (PSO) algorithm (Kennedy and Eberhart, 1995), artificial bee colony (ABC) algorithm (Karaboga, 2005), firefly algorithm (FA) (Yang, 2009), differential evolution (DE) (Storn and Price, 1997), genetic algorithm (GA) (Holland, 1975), whale optimization algorithm (WOA) (Mirjalili and Lewis, 2016), bat algorithm (BA) (Yang, 2010), salp swarm algorithm (SSA) (Wang et al., 2017), and flower pollination algorithm (FPA) (Yang, 2012). Kaya (2022) designed quick FPA algorithm training feedforward neural network (FNN) for solving parity problems. Pradeepkumar and Ravi (2017) used PSO algorithm to train the parameters of quantile regression neural network (QRNN) to predict volatility from financial time series. Asteris and Nikoo (2019) combined the ABC algorithm with FNN to predict the vibration period of infilled framed structures. Han et al. (2019) proposed a new model (DE-BP) for predicting cooling efficiency of forced-air precooling systems. Ding et al. (2013) applied GA to optimize the parameters of the Elman neural network, and experimentally verified that the convergence rate and training speed of the model were improved. Li et al. (2019) proposed an extreme learning machine based on WOA algorithm for aging assessment of Insulated gate bipolar transistor modules. Yang et al. (2016) designed a combinatorial model combining DE, BP neural network and adaptive network-based fuzzy inference system (ANFIS), and verified the effectiveness of the hybrid model on the electricity demand forecasting problem. Aljarah et al. (2018a) proposed a new method for optimizing FNN weights by the WOA algorithm. As with other models, the goal of RBFNNs in classification and regression problems is to maximize the classification rate and minimize the error of regression, which is equivalent to metaheuristic algorithm solving the global optimal value problem of the fitness function. Meanwhile, in order to verify the computational performance, practicality and persuasiveness of the hybrid model of metaheuristic algorithm and RBFNNs, some studies applied it not only to traditional classification and regression examples, but also to real problems. For example, Han et al. (2021) performed membrane bioreactor prediction with the help of the accelerated gradient algorithm. Han et al. (2022) introduced the accelerated second-order learning algorithm, and verified the accuracy of the model on issues such as prediction of water quality, nonlinear function approximation and system identification. Hu et al. (2018) proposed improved PSO-RBF and applied it to the operation cost prediction. These applied studies focus on areas that are especially needed in real life, bringing real benefits and outcomes to the economic development. Korürek and Doğan (2010) proposed an ECG beat classification method based on PSO and RBFNN. Jing et al. (2019) proposed the RBF-GA model for structural reliability analysis. Pazouki et al. (2022) designed a hybrid model of FA algorithm and RBFNN to predict the compressive strength of self-compacting concrete. Zhang and Wei (2018) developed improved PSO algorithm and RBFNN to forecast network traffic. Agarwal and Bhanot (2018) utilized FA algorithm to optimize the center vector in the hidden layer neurons of RBFNN and apply it to face recognition. Furthermore, BWO algorithm has the advantages of reliability and fast convergence speed, which promote its wide application in various fields. Micev et al. (2020) proposed adaptive black widow optimization algorithm to estimate synchronous machine parameters. Mukilan and Semunigus (2021) used deep convolution neural network (DCNN) based on the enhanced BWO algorithm for human object detection. The BWO algorithm was also used to optimize parameters for the ANFIS, SVR, and SVM (Katooli and Koochaki, 2020; Memar et al., 2021; Panahi et al., 2021). The application of the BWO algorithm is mainly in optimizing the parameters of neural networks. Analyzing the mathematical model of these algorithms, it is known that adaptive time-varying weight plays a crucial role in training network parameters. Therefore, this paper adopts the improved black widow optimization algorithm with time-varying inertia weight to learn RBFNNs to improve the accuracy and parsimony of the model, and uses its self-adjustment mechanism of local search ability and global search ability to optimize the parameters of RBFNNs. The proposed improved black widow optimization algorithm (IBWO)-RBF model is used for UCI dataset classification, nonlinear function approximation, nonlinear system identification and power load prediction.

The IBWO-RBF model combines the strengths of IBWO algorithm and RBFNNs. Therefore, the model can be applied to non-linear unstable time series forecasting, such as photovoltaic power forecasting, stock trend forecasting and water level forecasting. At present, the field of power load forecasting is of great significance in grid planning due to the connection of various new loads to the grid (Guo et al., 2020). Forecasting methods are divided into: traditional predictive models, machine learning models and combined models (Hermias et al., 2017). A single machine learning method is prone to reduce the accuracy of prediction results due to parameter limitations. Therefore, this paper uses the hybrid IBWO-RBF model to avoid defects such as weak generalization ability and large prediction error. Dong et al. (2017) proposed a method combining CNN and K-means clustering for short-term load forecasting. Ertugrul (2016) developed a recurrent extreme learning machine (RELM) method to predict electricity load. Hu et al. (2022) applied improved grasshopper optimization algorithm (IGOA), and long short-term memory (LSTM) network to forecast short-term load. Bashir et al. (2022) applied prophet-LSTM network based on BPNN to this field. Zhang et al. (2018) used fruit fly optimization algorithm (FOA) to train a hybrid model for power load forecasting.

Most studies use metaheuristic algorithms to optimize neural network parameters and verify their effectiveness on classification or regression problems. However, it has not been verified that the same model has good results in both classification and regression. Therefore, in this study, the proposed model will be tested on both classification and regression problems to verify its effect. What calls for special attention is that many metaheuristic algorithms [PSOGSA (Mirjalili et al., 2012), ABWO (Liu et al., 2022)] considered linear time-varying inertia weight to escape from the local optimum. Time-varying inertial weight of metaheuristic algorithm is the key to training RBFNNs. Therefore, in order to achieve greater accuracy in several classification and regression problems, this article improves the random inertia weight to nonlinear time-varying inertia weight. The IBWO algorithm is proposed. On the basis of the existing linear time-varying inertia weight of ABWO algorithm, nonlinear time-varying inertia weight is designed, so that the effect of IBWO algorithm optimizing RBFNNs is more significant. The IBWO algorithm is combined with RBFNNs. The IBWO-RBF model and other models compare and analyze the results on classical dataset classification, nonlinear function approximation, nonlinear system identification and real-life power load prediction problems. The innovations and contributions of this paper are summarized as follows:

(1) This article develops the IBWO-RBF model. Experiments illustrated the excellence of the proposed model in several aspects. Most articles compare the effectiveness of methods in classification or regression. However, the IBWO-RBF model in this paper verifies both the classification performance and the regression effect.
(2) This paper proposes the IBWO algorithm. Consulting many literatures shows that the time-varying inertia weights of metaheuristic algorithms are the key to training RBFNNs. Therefore, nonlinear time-varying inertia weights are designed to make the optimization of RBFNNs more effective.
(3) The proposed model is not only superior to other models on several classical problems, but also performs well in practical problem. The IBWO-RBF model is a new and effective approach to solve the problem of short-term power load forecasting, providing a priori knowledge for the safe operation and planning of the power grid.

The remainder of this paper is summarized as follows: section “2 Proposed model” briefly describes the structure of IBWO algorithm, RBFNN, and IBWO-RBF model. The simulation results of the IBWO-RBF model on traditional classification and regression examples are introduced and discussed in section “3 Experiments on common classification and regression problems.” The performance of the IBWO-RBF model on power load prediction is presented and discussed in section “4 Experiments on real problems in short-term power load forecasting.” In section “5 Conclusion and future study” are presented.

## 2. Proposed model

### 2.1. RBF neural network (RBFNN)

**FIGURE 1:**
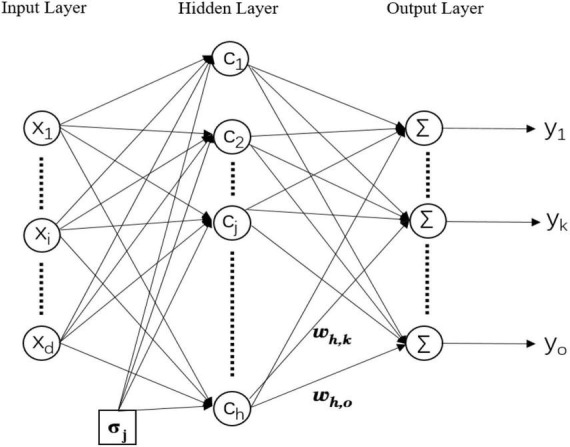
Multiple-input multiple-output radial basis function neural network (RBFNN).

The experiment used a three-layer RBFNN (input layer, hidden layer, and output layer), the specific structure of which is shown in Figure 1. The input layer is connected to the hidden layer through an activation function. The activation function in the hidden layer utilizes Gaussian kernel function, which is expressed in Equation (1). RBF networks are special form of multilayer feedforward neural networks (MLP) (Du and Swamy, 2006). In MLP, activation functions in the hidden neurons are sigmoidal functions, while in RBF networks the Gaussian basis functions are typically used (Mirjalili et al., 2012). Each hidden layer neuron of RBFNNs contains a Gaussian-based activation function that measures the similarity between samples and samples. The reason why the Gaussian basis function has been exploited with respect to other activation functions is that the Gaussian kernel function maps each sample point to an infinite-dimensional feature space, so that the otherwise linearly indivisible data becomes linearly separable. The relationship between hidden layer and output layer is described in Equation (2).


(1) $$h_{j}=e^{-{∥x-c_{j}∥}^{2}\,/\,2\,\sigma_{j}^{2}}$$

(2) $$y_{k}=\sum_{j=1}^{h}w_{j,k}\,h_{j}$$

where *d*, *h*, and *o* are the number of neurons in the input layer, hidden layer, and output layer, respectively. *x*_*i*_,*h*_*j*_, and *y*_*k*_ represent the *i*th neuron in the input layer, the *j*th neuron in the hidden layer, and the *k*th neuron in the output layer. The input vector is*x* = [*x*_1_,*x*_2_,…,*x*_*d*_]^T^. *w*_*j*,*k*_ is the output weight between the *j*th hidden neuron and the *k*th output neuron. *c*_*j*_ is the center vector of the *j*th hidden neuron. σ_*j*_ is the width of the *j*th hidden neuron. ∥*x*−*c*_*j*_∥ is the Euclidean distance between the input vector and the center vector. The topological size of RBFNN is determined by the number of hidden layer neurons.

### 2.2. IBWO algorithm for training RBFNN

Swarm intelligence algorithm is a kind of metaheuristic algorithm, which is an optimization method with complete theory and strong optimization performance. Similar to other swarm intelligence algorithms, the BWO algorithm (Hayyolalam and Kazem, 2020) searches for optimal solution based on the reproductive behavior of black widow spiders (crossover, cannibalism, and mutation), which has the advantages of fast convergence speed and high optimization accuracy. The search steps of the BWO algorithm are described as follows:

Step 1. The BWO algorithm initializes a random spider population. Each spider individual is represented by a candidate solution vector of the algorithm, i.e., [b_1_,*b*_2_,…,*b*_dim_].
Step 2. The fitness function of the problem to be solved is designed to calculate the quality of the solution. Since strong offspring survive, candidate solutions with smaller fitness function values are preserved. The better solution is retained and the worse solution is deleted.
Step 3. Crossover behavior is the process by which both parents produce offspring according to the crossover rate. The mathematical model corresponding to the crossover behavior is shown in Equation (3).

(3) $$\left\{ \begin{array}{c} y_{1}=\alpha .^{*}b_{1}+\left(1-\alpha \right).^{*}b_{2}\\ y_{2}=\alpha .^{*}b_{2}+\left(1-\alpha \right).^{*}b_{1} \end{array}\right.$$

**FIGURE 2:**
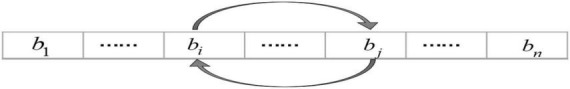
The mutation behavior of a spider individual.

Where each element of the α array is a random value in the range [0,1]. *b*_1_ and *b*_2_ are parents. *y*_1_ and *y*_2_ are descendants. The crossover process is repeated dim⁡/2 times. Cannibalism refers to the fact that in both parents, strong female spiders eat weak male spiders; Among offspring, strong offspring eat weak offspring. Mutation behavior is the process by which the original individual produces a new individual according to the mutation rate. The specific mutation process is shown in Figure 2, e.g., [*b*_1_,…,*b*_*i*_,…,*b*_*j*_,…,*b*_*n*_] mutates to [*b*_1_,…,*b*_*j*_,…,*b*_*i*_,…,*b*_*n*_]. This step is to search for other individuals throughout the search space, enhance population diversity, and update candidate solutions.

Step 4. Cycle through step 2 and step 3. Candidate solutions are continuously updated and fitness function values are compared until the maximum number of iterations is reached or converges to the optimal value.

Although excellent performance was reported, BWO algorithm has the potential to suffer from premature convergence to a point. Inspired by Fan and Chiu (2007), the β array is no longer a random value, but changes nonlinearly over time. The equation for the β array of the IBWO algorithm is as follows:


(4) $$\beta_{i}=8\times {\left(\frac{2}{\text{iter}}\right)}^{0.3}\,\beta =\left[\beta_{1},\beta_{2},\ldots ,\beta_{n}\right]$$

Therefore, the update equation for the crossover process is:


(5) $$\left\{ \begin{array}{c} y_{1}=\alpha .^{*}b_{1}+\left(1-\alpha \right).^{*}b_{2}\\ y_{2}=\beta .^{*}b_{2}+\left(1-\beta \right).^{*}b_{1} \end{array}\right.$$

where *b*_1_ and *b*_2_ are parents. *y*_1_ and *y*_2_ are descendants. *iter* is the current number of iterations. Experiments had shown that the constant of the β array is set to 8, which makes the global search ability of the algorithm stronger.

### 2.3. System architecture of the IBWO-RBF neural network

**FIGURE 3:**
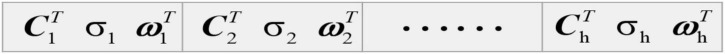
The structure of each black widow individual.

The IBWO algorithm simultaneously searches the center vector, width, and output vector of RBFNN. The IBWO algorithm and RBFNN are connected by candidate solution vectors of the IBWO algorithm, which is shown in Figure 3.

where *c*_*j*_, σ_*j*_, and *w*_*j*_ are the center vector, width, and output vector of the *j*th hidden neuron for each black widow individual, respectively. The number of hidden layer neurons in the IBWO-RBF model determines the size of the network. Therefore, *h* is both the number of hidden layer neurons and the size of the neural network. The dimension of the black widow is determined by the number of neurons in the input layer, hidden layer and output layer of IBWO-RBF. *D* is the dimension of the black widow, and *D* is equal to *d* + 1 + *o*. *d* is the number of input vector and *o* is the number of output vector. The number of input layer neurons of IBWO-RBFNN is determined by the dimension of the input data. The model is a single-output model when solving regression problems, but a multiple-output model when solving classification problems. The number of neurons in the output layer of IBWO-RBFNN is determined by the number of categories of classification problems.

**FIGURE 4:**
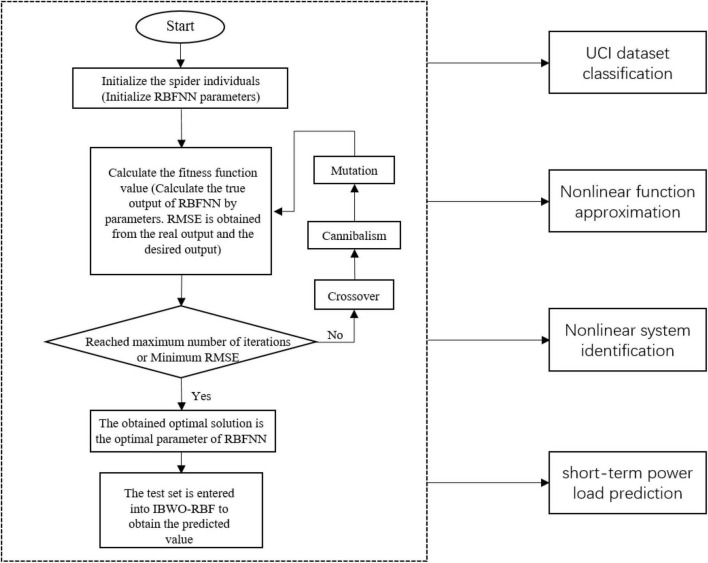
System architecture of the improved black widow optimization algorithm (IBWO)-RBF model.

For classification and regression problems, Root Mean Square Error (RMSE) is used as a fitness function to optimize RBFNN. Take the classification problem as an example, the specific calculation equation is expressed as follows. The RMSE is expected to be as close to 0 as possible. Consequently, the goal of the search process is to obtain an optimal solution that minimizes the fitness function value. The global optimal widow optimized by the IBWO algorithm is the optimal parameter of RBFNN. Figure 4 shows the overall architecture of this article.


(6) $$R\,M\,S\,E=\sqrt{\frac{1}{N}\,\sum_{k=1}^{N}{\left(y_{k}-f\,\left(x_{k}\right)\right)}^{2}}$$

where *N* is the number of output neurons, *y*_*k*_ and *f*(*x*_*k*_) are the real output of the model and the desired output, respectively.

## 3. Experiments on common classification and regression problems

### 3.1. Classification problems for UCI datasets

In this section, the parameters of the RBFNN were trained using the IBWO algorithm for a classification problem on 10 binary classification datasets. And IBWO was compared with 12 algorithms, namely PSO, GA, ABC, DE, FA, CS, BA, ABWO, BWO, biogeography-based optimization algorithm (BBO), ant clony optimization algorithm (ACO), and gradient-based algorithm (GB).

#### 3.1.1. Experimental setup

The 10 binary classification datasets used in this section were selected from the UCI repository, and the detailed descriptions are shown in Table 1. All datasets are trained and tested at 66 and 34%, respectively. The population size of all algorithms was fixed at 50, and each classification experiment was run 10 times independently, with 250 iterations for each run.

**TABLE 1:** Description of the classification datasets.

Datasets	No. of features	No. of training samples	No. of testing samples
Australian	14	455	235
Blood	4	493	255
Breast	8	461	238
Diabetes	8	506	262
Diagnosis I	6	79	41
German	24	660	340
Diagnosis II	6	79	41
Hepatitis	10	102	53
Liver	6	227	118
Vertebral	6	204	106

Without prior knowledge, the number of hidden layer neurons is not easy to determine (Wang et al., 2019). In order to reduce the error caused by the randomness of the neural network, this paper adopted the trial-and-error method (Kermanshahi, 1998) to determine the number of hidden layer neurons. When the number of hidden layer neurons was set from 3 to 16 in IBWO-RBF, set to 4, 6, 8, and 10 in all other algorithms. All algorithms selected the structure of IBWO-RBF with the best classification result, and then obtained the corresponding number of hidden neurons. The specific operation is as follows: first, the parameters of the neural network were arbitrarily given, and each subsequent selection only changed the number of hidden neurons. Under the condition that the other parameters remained unchanged, repeated experiments were repeated 10 times, and by comparing the obtained average classification rates, the number of hidden neurons with the highest average classification rate was finally selected.

#### 3.1.2. Data preprocessing

Some datasets downloaded from the UCI repository have abnormal problems, such as missing values, containing English characters, etc. Therefore, the dataset was preprocessed (including outlier handling and data normalization). First, the samples with missing information were deleted; second, the English strings were replaced with numbers such as 0, 1, 2, and 3; third, in order to eliminate the dimensional influence between the several features of the dataset, and to solve the data attributes comparability. Therefore, the data standardization process was carried out, and the attribute value and the classification value were normalized to [−1, 1]. Finally, the training set and testing set were randomly selected from the dataset samples in proportion. The attribute value of the training sample was used as the input of the model, and the category of the training sample was used as the output of the model.

#### 3.1.3. Experimental results and discussion

In this section, all models were compared in terms of accuracy (i.e., average classification rate) and structural complexity, and the Friedman test was carried out to obtain the ranking of the algorithms. Table 2 shows the average accuracy of 10 independent runs of 13 models on 10 datasets. Table 3 describes the number of hidden neurons that gave the best results. The best accuracy results and the least number of neurons are highlighted in bold. The proposed model achieved 81.02% classification accuracy on the Blood dataset, whereas GB-RBF achieved 81.18% accuracy. On the Blood classification dataset, the proposed model ranked second. On other datasets except the Blood dataset, the average classification rate of IBWO-RBF had obtained better results than all other algorithms, that is, ranked first. Not only that, it can be seen from Table 3 that on most datasets, the IBWO-RBF model used fewer neurons in the hidden layer than other models. Especially on the Hepatitis, Diagnosis I, Australian, Diagnosis II, German and Liver datasets, the IBWO-RBF model used the simplest structure and the least number of hidden neurons. The proposed IBWO-RBF model not only has high classification accuracy, but also has a simple network structure.

**TABLE 2:** Average classification rate (in %) of 13 algorithms on 10 datasets.

Datasets	IBWO	ABWO	BWO	BBO (Aljarah et al., 2018b)	GA (Aljarah et al., 2018b)	PSO (Aljarah et al., 2018b)	ACO (Aljarah et al., 2018b)
Blood	**81**.**75%**	81.02%	76.12%	77.45%	77.22%	76.90%	76.39%
Breast	**98**.**32%**	97.90%	88.28%	97.86%	96.89%	93.15%	68.53%
Hepatitis	**90**.**00%**	89.81%	81.13%	84.72%	84.72%	83.58%	66.42%
Diabetes	**77**.**94%**	77.56%	69.81%	71.60%	70.00%	67.52%	60.08%
Vertebral	**87**.**23%**	84.85%	70.00%	78.40%	77.08%	74.06%	64.53%
Diagnosis I	**100**.**0%**	100.0%	100.0%	100.0%	100.0%	86.34%	57.32%
Diagnosis II	**100**.**0%**	100.0%	91.95%	100.0%	100.0%	88.54%	57.32%
Liver	**72**.**37%**	70.17%	64.41%	69.15%	64.66%	58.98%	52.20%
German	**74**.**08%**	72.97%	71.18%	71.91%	68.09%	56.38%	55.91%
Australian	**88**.**13%**	85.60%	79.10%	85.32%	84.55%	71.57%	58.85%
Datasets	IBWO	DE (Aljarah et al., 2018b)	FA (Aljarah et al., 2018b)	CS (Aljarah et al., 2018b)	GB (Aljarah et al., 2018b)	ABC (Aljarah et al., 2018b)	BA (Aljarah et al., 2018b)
Blood	**81**.**75%**	76.63%	76.94%	77.18%	81.18%	76.63%	77.57%
Breast	**98**.**32%**	86.26%	96.60%	96.43%	96.22%	96.13%	97.44%
Hepatitis	**90**.**00%**	80.00%	85.47%	86.23%	83.02%	83.77%	85.09%
Diabetes	**77**.**94%**	63.78%	69.31%	69.54%	77.48%	69.89%	71.18%
Vertebral	**87**.**23%**	70.19%	75.75%	75.66%	83.02%	72.64%	77.83%
Diagnosis I	**100**.**0%**	70.24%	98.05%	98.78%	100.0%	92.44%	100.0%
Diagnosis II	**100**.**0%**	67.07%	97.07%	100.0%	100.0%	95.37%	100.0%
Liver	**72**.**37%**	56.10%	61.69%	62.29%	61.02%	57.20%	65.68%
German	**74**.**08%**	52.65%	61.29%	66.82%	72.65%	67.12%	67.26%
Australian	**88**.**13%**	60.64%	76.94%	79.45%	85.11%	80.64%	83.62%

Optimal values are given in bold.

**TABLE 3:** Minimum number of hidden neurons for 13 algorithms on 10 datasets.

Datasets	IBWO	ABWO	BWO	BBO	GA	PSO	ACO	DE	FA	CS	GB	ABC	BA
Blood	5	9	**3**	8	8	6	10	10	6	4	10	10	10
Breast	14	12	5	10	8	8	10	8	10	8	6	**4**	6
Hepatitis	5	8	**3**	6	10	4	6	4	4	4	4	10	4
Diabetes	**4**	9	**4**	10	10	8	**4**	**4**	6	8	8	6	6
Vertebral	10	9	9	8	10	**4**	6	6	10	10	6	6	10
Diagnosis I	**3**	**3**	**3**	4	6	4	6	6	4	8	8	4	8
Diagnosis II	**3**	**3**	4	4	6	8	4	10	4	4	4	4	10
Liver	4	**3**	8	10	8	8	10	4	4	4	10	8	8
German	**3**	4	4	10	6	4	8	8	10	4	8	4	10
Australian	**3**	**3**	**3**	4	10	4	6	4	6	6	4	4	8

Optimal values are given in bold.

Additionally, to make this conclusion convincing, a Friedman statistical test was completed to test the significance of the average classification rates. The Friedman test obtained the ranking of 14 optimized algorithms by analyzing the average accuracy value of each dataset. The sorted table is shown in Table 4. It can be known that the IBWO-RBF model ranks first. Sensitivity and specificity of these algorithms on 10 datasets is shown in Table 5. Optimal sensitivity and specificity are highlighted in bold. Compared with the other 12 models, the proposed model showed stronger sensitivity and specificity on 9 datasets (Blood, Breast, Hepatitis, Diabetes, Vertebral, Diagnosis I, Diagnosis II, Liver, and Australian). It can be seen that the IBWO algorithm achieves higher classification accuracy on each dataset than the original BWO algorithm. Therefore, the improvement to the original BWO algorithm in this paper greatly improve its classification performance. Combining the above experimental results, it can be clearly concluded that IBWO outperforms most other algorithms in optimizing the parameters of RBFNNs for classification problems, which proves the strength of the IBWO algorithm in training RBFNNs.

**TABLE 4:** Friedman test results.

Algorithm	Friedman rank	General rank
ABWO	2.4500	2
IBWO	**1**.**8500**	**1**
ACO	12.8500	13
BWO	7.2000	7
BBO	4.2500	3
GA	5.7500	6
PSO	10.3000	11
DE	11.7000	12
FA	8.0500	9
CS	7.2500	8
GB	5.0500	4
ABC	9.1000	10
BA	5.2000	5

Optimal values are given in bold.

**TABLE 5:** Sensitivity and specificity of these algorithms on 10 datasets.

Datasets	IBWO	ABWO	BWO	BBO	GA	PSO	ACO	DE	FA
Blood	0.9899	0.9898	**1.0000**	0.9979	0.9969	0.9969	0.8574	0.9908	0.9985
Breast	0.9804	0.9936	**1.0000**	0.9790	0.9720	0.9656	0.7261	0.9191	0.9732
Hepatitis	**0.6870**	0.5969	0.0000	0.6300	0.5600	0.2100	0.4300	0.2300	0.3800
Diabetes	0.9036	0.8869	**0.9625**	0.4354	0.3063	0.3469	0.3052	0.1438	0.3104
Vertebral	0.9102	0.9812	**1.0000**	0.8973	0.8920	0.8773	0.7453	0.8760	0.8667
Diagnosis I	**1.0000**	**1.0000**	**1.0000**	**1.0000**	**1.0000**	0.9474	0.5000	0.6053	0.9579
Diagnosis II	**1.0000**	**1.0000**	0.9262	**1.0000**	**1.0000**	0.9591	0.5545	0.6591	0.9955
Liver	0.2842	0.2865	0.0000	**0.3500**	0.2700	0.1880	0.3360	0.2020	0.3020
German	0.9907	0.9920	**1.0000**	0.9518	0.9662	0.5877	0.6596	0.5662	0.7925
Australian	0.9031	0.8666	**0.9755**	0.8634	0.8218	0.5356	0.6307	0.5356	0.6287
Datasets	IBWO	ABWO	BWO	BBO	GA	PSO	ACO	DE	FA
Blood	**0.1667**	0.1201	0.0000	0.0483	0.0417	0.0283	0.1583	0.0200	0.0250
Breast	**0.9916**	0.9445	0.6257	0.9519	0.9630	0.8654	0.3593	0.7531	0.8938
Hepatitis	0.9568	0.9619	**1.0000**	0.8744	0.9116	0.9767	0.7116	0.7791	0.9395
Diabetes	0.5497	0.5784	0.1394	0.8524	**0.9145**	0.8651	0.7211	0.9054	0.9036
Vertebral	**0.7444**	0.3136	0.0000	0.5097	0.4484	0.3581	0.3226	0.1710	0.4742
Diagnosis I	**1.0000**	**1.0000**	**1.0000**	**1.0000**	**1.0000**	0.6000	0.5455	0.5864	0.9364
Diagnosis II	**1.0000**	**1.0000**	0.9048	0.9895	**1.0000**	0.8000	0.4789	0.6000	0.9000
Liver	0.9334	0.9323	**1.0000**	0.8824	0.9235	0.8853	0.6559	0.7941	0.7941
German	0.0077	0.0212	0.0000	0.2295	0.0911	**0.4902**	0.3545	0.4455	0.2116
Australian	**0.8515**	0.8170	0.5595	0.8313	0.8470	0.6851	0.3948	0.6030	0.8239

Optimal values are given in bold.

### 3.2. Nonlinear regression problems

In this section, the IBWO-RBF model was used in nonlinear function approximation and nonlinear system identification, two nonlinear regression problems, and compared with five models, namely the BWO-RBF model, the Euclidean kernel based RBF (EK-RBF), the Cosine kernel based RBF (CK-RBF), the dynamic fusion of Euclidean and cosine kernels based RBF (DF-RBF) and the manual fusion of Euclidean and cosine kernels based RBF (MF-RBF). The results of the comparison were converted by the following equation and expressed in one electrical unit (dB).


(7) a=10⁢log⁡(a)⁢d⁢B

The evaluation index is the mean square error (MSE), which is described as follows:


(8) $$M\,S\,E=\frac{1}{n}\,\sum_{j=1}^{n}{\left(y_{j}-f\,\left(x_{j}\right)\right)}^{2}$$

where *n* is the number of samples, *y*_*j*_ and *f*(*x*_*j*_) are the real output of the network and the desired output, respectively.

#### 3.2.1. Nonlinear function approximation

The nonlinear function approximation took the following equation as an example:


(9) $$f\,\left(x,y\right)=e^{\left(x^{2}-y\right)}$$

**FIGURE 5:**
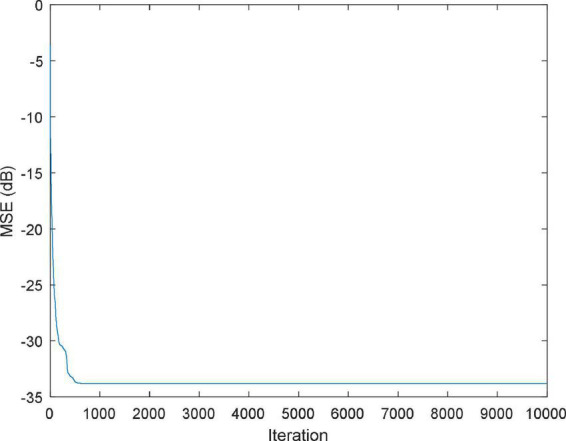
MSE value of the improved black widow optimization algorithm (IBWO)-RBF.

**FIGURE 6:**
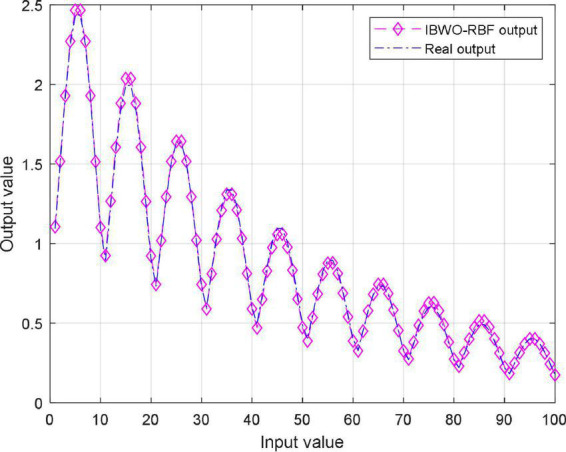
Approximation results of the improved black widow optimization algorithm (IBWO)-RBF.

The function *f*(*x*,*y*) is approximately replaced by a simple function P(*x*,*y*) with the aim of minimizing the error of P(*x*,*y*) and *f*(*x*,*y*) in a metric sense (Zhang Y. et al., 2022). Figures 5, 6 shows the MSE value and approximation results of the IBWO-RBF model on the approximation of a nonlinear function, respectively. The MSE and the number of hidden layer neurons compared with the other four models are described in Table 6.

**TABLE 6:** Comparison of six models for the approximation of nonlinear function.

Model	Testing MSE (dB)	No. of hidden neuros
IBWO-RBF	-**33**.**9305**	**4**
BWO-RBF	-4.5217	7
EK-RBF (Khan et al., 2017)	-18.6619	121
CK-RBF (Khan et al., 2017)	-4.9277	121
DF-RBF (Khan et al., 2017)	-18.4076	121
MF-RBF (Khan et al., 2017)	-15.6181	121

Optimal values are given in bold.

It can be seen that the MSE of the IBWO-RBF model is the smallest, which is −49.8444dB, and the number of hidden neurons is the least, and the neural network structure is simpler. Again, under the same number of iterations of 10,000 for the five models, the IBWO-RBF model converges faster and achieves early convergence. It is evident from Figure 6 that the proposed model fits the points on the nonlinear function perfectly. Moreover, the model converged to the optimal value within only 1,000 generations. Both function approximation accuracy and search speed show excellent results. Experiments show that the IBWO-RBF model outperforms the other four variants of RBFNNs and BWO-RBF model in both the accuracy of function approximation and the complexity of the network structure.

#### 3.2.2. Nonlinear system identification

**FIGURE 7:**
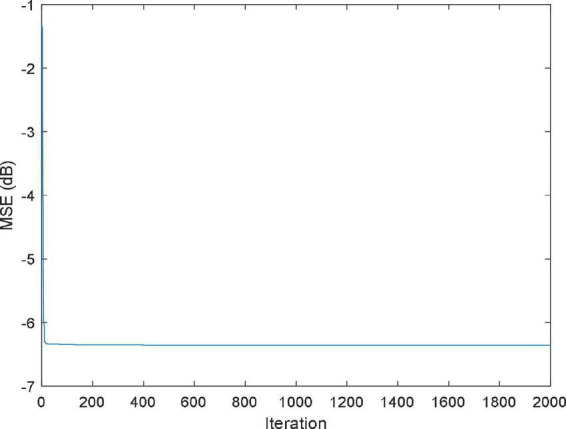
MSE value of the improved black widow optimization algorithm (IBWO)-RBF against iterations.

**FIGURE 8:**
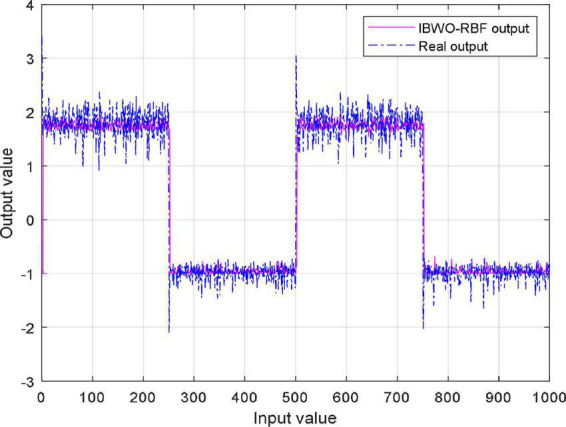
Identification results of the improved black widow optimization algorithm (IBWO)-RBF model.

The nonlinear system is given by the following equation:


y⁢(t)=2⁢r⁢(t)-0.5⁢r⁢(t-1)-0.1⁢r⁢(t-2)-0.7

(10) $$\left[\cos\left(3\,r\,\left(t\right)\right)+e^{-\left|r\,\left(t\right)\right|}\right]+n\,\left(t\right)$$

where *r*(*t*) is the input of the system, and *y*(*t*) is the output; the model is assumed to be N(0,0.0025), *n*(*t*) is the disturbance of the system; the constants are the polynomial coefficients. Figure 7 illustrates the MSE values of the IBWO-RBF model for nonlinear system identification. Moreover, Identification results are shown in Figure 8. The MSE and the number of hidden layer neurons compared with the other four models are shown in Table 7. It can be seen from the above figure that the IBWO-RBF model converges within 100 iterations, which is faster than the other five models and has a smaller MSE. The MSE of the proposed model is much smaller than that of the original BWO algorithm combined with RBFNN. Moreover, the conclusions support the advantage of the proposed model for the nonlinear system identification with smaller network structures.

**TABLE 7:** Comparison of six models for the nonlinear system identification.

Model	Testing MSE (dB)	No. of hidden neuros
IBWO-RBF	-**8**.**3535**	**4**
BWO-RBF	-2.0475	4
EK-RBF (Khan et al., 2017)	-6.1943	401
CK-RBF (Khan et al., 2017)	2.7887	401
DF-RBF (Khan et al., 2017)	-6.1547	401
MF-RBF (Khan et al., 2017)	-5.5176	401

Optimal values are given in bold.

## 4. Experiments on real problems in short-term power load forecasting

### 4.1. Data description

The dataset in this section is from the National Collegiate Electrotechnical Mathematical Modeling Competition.^1^ The dataset is from 1 January to 14 May 2012 with a time interval of 15 min, including meteorological factors such as daily maximum temperature, daily minimum temperature, daily average temperature, daily relative humidity, and daily rainfall. The experiment selected data from 0:00 to 23:00 with an interval of 1 h. To improve forecast accuracy, historical data that is less relevant to the meteorological data on the forecast date was removed. A set of highly correlated meteorological data remained, for a total of 50 days. The multiple-input single-output IBWO-RBF model was adopted, which had 25 neurons in the input layer, 4 neurons in the hidden layer, and 1 neuron in the output layer. For 0:00, the input for each training sample was the load value of 0:00 for the previous 20 days and the meteorological data for the next day, and the output was the load value of 0:00 for the next day. The input of the test sample was the load value of 0:00 for the 20 days before the forecasting day, and the meteorological data for the forecasting day. The output of the test sample was the load value of 0:00 for the forecasting day. The experimental method at other time points was the same as 0:00 (Chen et al., 2018). The IBWO-RBF model was compared to the other 5 models.

### 4.2. Experimental setup

The Matlab R2019a is used to implement IBWO-RBF, BWO-RBF, PSO-RBF, RBF, ELM, and BPNN. All models are run independently 10 times to take the average. The number of iterations and population size for all algorithms are 500 and 50, respectively. For the IBWO-RBF and BWO-RBF model, the crossover rate is 0.8, the mutation rate is 0.4, and the cannibalism rate is 0.5. Acceleration constants of the PSO-RBF model is [2, 2], and inertia weights is [0.9, 0.6]. For the three hybrid models (IBWO-RBF, BWO-RBF, and PSO-RBF), the number of hidden layer neurons in each model is 4. A single RBF neural network model is designed quickly and error-free using the newrbe function. The number of hidden layer neurons defaults to the number of samples in the training set, which is set to 5 in the ELM model. Similarly, the number of hidden layer neurons in the BPNN model is also set to 5.

### 4.3. Experimental results and discussion

The predicted values and prediction errors are shown in Tables 8, 9, respectively. From 00:00 to 23:00, the smallest error at each time is marked in bold. The RMSE for IBWO-RBF, BWO-RBF, PSO-RBF, RBF, ELM and BPNN were 357.8041, 565.9603, 589.0443, 1,220.5, 666.3963, and 576.4008, respectively. The proposed model has the smallest RMSE, and the results of the BWO-RBF model are second only to the proposed model. In addition to 7:00, 8:00, 16:00, and 19:00, compared with the other three single models, IBWO-RBF, BWO-RBF, and PSO-RBF obtained the best predicted values. It can be seen that the hybrid model of metaheuristic algorithm and RBFNN is better than that of a single model, which verifies the effectiveness of the combinatorial method. Most of the time, the IBWO-RBF model has less error than all other models. In addition to 6:00 and 7:00, the IBWO-RBF model has a smaller error than the BWO-RBF, indicating that improvements to the IBWO algorithm can also improve the prediction accuracy of real-world problems.

**TABLE 8:** Comparison of predicted values of 6 models.

Model	00:00	01:00	02:00	03:00	04:00	05:00	06:00	07:00
IBWO-RBF	5,426.69	5,110.07	4,794.636	4,649.68	4,611.14	4,484.95	4,505.54	4,693.12
BWO-RBF	5,153.87	4,900.55	4,716.7072	4,573.61	4,471.50	4,418.80	4,450.309	4,689.98
PSO-RBF	5,418.15	5,130.61	4,955.097	4,799.39	4,671.10	4,614.82	4,591.087	4,698.79
RBF	5,180.91	4,788.97	4,606.573	4,887.49	4,769.65	4,710.39	4,703.555	4,962.91
ELM	5,048.66	4,715.81	5,137.183	4,577.13	4,741.40	4,647.35	4,568.974	4,683.05
BPNN	5,166.99	4,831.32	5,128.448	4,558.24	4,934.49	4,172.36	4,783.941	4,751.53
Model	08:00	09:00	10:00	11:00	12:00	13:00	14:00	15:00
IBWO-RBF	5,987.28	6,996.63	7,009.111	7,478.95	7,170.72	5,603.31	6,913.8937	7,116.63
BWO-RBF	5,997.63	7,305.10	7,535.05	7,713.86	7,553.48	5,798.63	7,262.1758	7,355.08
PSO-RBF	6,040.88	7,357.75	7,626.29	7,820.53	7,634.47	5,844.09	7,325.705	7,336.06
RBF	6,519.97	8,524.77	8,960.290	9,154.71	7,886.27	6,763.15	8,471.895	8,616.72
ELM	6,094.19	7,303.57	7,764.357	7,932.83	7,709.91	5,996.93	7,410.4954	7,570.00
BPNN	5,705.65	5,655.16	7,529.958	7,790.99	7,403.92	6,036.73	5,679.8933	7,462.45
Model	16:00	17:00	18:00	19:00	20:00	21:00	22:00	23:00
IBWO-RBF	7,480.56	7,239.33	6,192.553	7,013.47	6,713.56	6,806.76	6,400.516	5,696.99
BWO-RBF	7,675.84	7,566.07	6,445.065	7,179.26	7,149.20	6,989.55	6,628.993	5,789.39
PSO-RBF	7,729.58	7,692.51	6,464.862	7,011.55	7,098.27	7,013.38	6,660.552	5,883.32
RBF	6,922.04	8,840.94	7,384.90	7,310.35	7,188.52	7,070.37	6,832.366	6,419.53
ELM	7,868.79	7,706.05	6,582.879	7,178.60	7,033.25	7,187.82	6,553.411	5,936.75
BPNN	8,317.44	7,965.29	6,807.712	7,506.18	7,274.94	7,544.36	7,122.221	6,347.74

**TABLE 9:** Comparison of prediction errors of 6 models.

Model	00:00	01:00	02:00	03:00	04:00	05:00	06:00	07:00
IBWO-RBF	44.1343	**9**.**27006**	84.1806	**67**.**2371**	**41**.**6170**	**18**.**23**	44.5362	150.957
BWO-RBF	228.681	200.248	162.1096	143.309	98.0263	84.3879	**10**.**692**	147.817
PSO-RBF	**35**.**5957**	29.8098	**76**.**2805**	82.3184	101.573	111.640	130.0854	156.620
RBF	201.637	311.824	272.2432	170.567	200.120	207.208	242.5536	420.747
ELM	333.891	384.987	258.3658	139.784	171.870	144.165	107.9732	140.887
BPNN	329.387	359.903	157.4553	236.349	260.970	414.915	298.5727	**138**.**169**
Model	08:00	09:00	10:00	11:00	12:00	13:00	14:00	15:00
IBWO-RBF	533.148	**544**.**983**	**220**.**299**	**499**.**466**	**321**.**584**	**102**.**410**	**369**.**77135**	**468**.**618**
BWO-RBF	543.497	853.457	746.236	734.385	704.341	297.731	718.0535	707.065
PSO-RBF	586.751	906.108	837.483	841.049	785.334	343.195	781.5824	688.048
RBF	1,065.84	2,073.13	2,171.48	2,175.23	1,037.13	1,262.26	1,927.7723	1,968.71
ELM	640.061	851.926	975.545	953.345	860.779	496.038	866.37314	921.991
BPNN	**251**.**513**	796.490	741.148	811.512	554.789	535.834	864.22897	814.434
Model	16:00	17:00	18:00	19:00	20:00	21:00	22:00	23:00
IBWO-RBF	517.835	**377**.**466**	**250**.**8334**	761.395	**417**.**195**	**535.060**	**348**.**1445**	**218**.**582**
BWO-RBF	713.121	704.210	503.3449	927.188	852.835	717.847	559.3505	310.981
PSO-RBF	766.863	830.640	523.1417	759.473	801.905	741.674	608.1812	404.907
RBF	**40**.**6773**	1979.07	1,443.18	1,058.28	892.152	798.665	779.9946	941.115
ELM	906.069	844.190	641.1587	926.527	736.884	916.118	501.0396	458.342
BPNN	576.133	414.426	404.055	**705**.**833**	457.629	822.533	690.5537	477.315

Optimal values are given in bold.

**FIGURE 9:**
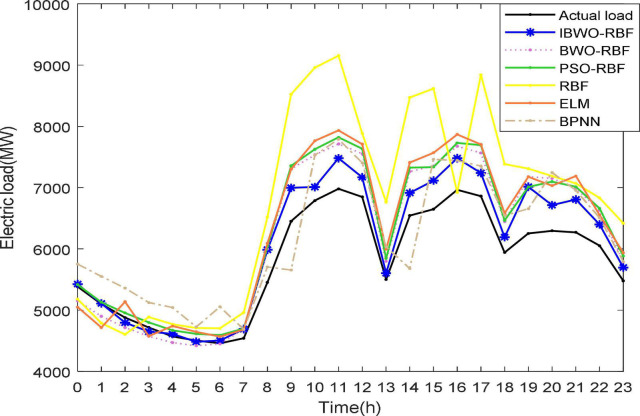
Forecasting curves of 6 models and real load curves.

**FIGURE 10:**
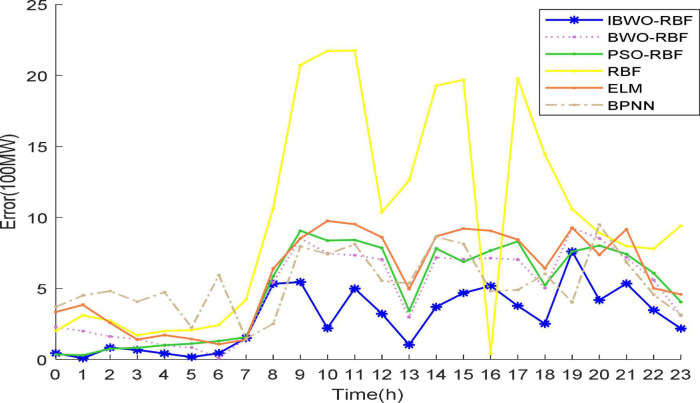
Predicted error curves of 6 models.

Again, Figures 9, 10 more vividly demonstrate the predictive performance of the 6 models. All models can predict approximate curves close to the true load values. It can be clearly seen in the Figure 10, from 22:00 to 7:00, all models show better performance. The reason for this phenomenon may be that less electricity is used at night, resulting in more stable predictions, while higher electricity consumption during the day causes inaccurate predictions. It indicates that the load value at night is more accurately predicted. Compared to other models, from 9 to 23 o’clock, the predicted values of the proposed model are more accurate. During the day, the proposed model stands out more than other models for predictive performance. A single RBFNN performed the worst, but combining RBFNN with algorithms outperformed ELM and BPNN. The curve of the IBWO-RBF model is closest to the curve of the true load value, and the error curve of the model is mostly at the bottom. Therefore, the proposed model has the smallest prediction error, the highest optimization accuracy, and its prediction curve is closest to the expected value curve. After experimental verification, the prediction performance of the proposed model for the actual power load problem is worthy of recognition.

## 5. Conclusion and future work

A novel hybrid model called IBWO-RBF is applied for solving classical dataset classification, nonlinear function approximation, nonlinear system identification, and real power load prediction. The IBWO algorithm introduces a nonlinear time-varying factor to better balance the diversity and convergence for optimizing parameters of a RBFNN. First, IBWO-RBF classifies some classification datasets in the UCI repository. The conclusion proves that IBWO-RBF has higher classification accuracy and simpler structure than metaheuristic algorithms such as PSO, GA, DE, and so on. Again, the proposed model also converges faster than other RBFNNs on nonlinear learning problems with higher optimization accuracy. In addition, compared with other models, the proposed model proves its superior power load prediction performance. The experimental results demonstrated obviously that the developed model is effective, competitive and promising tool for solving classification and nonlinear regression problems. In the future, this model can be developed in power plants to predict power load simply and efficiently. Furthermore, metaheuristic algorithms can be combined with deep learning networks to solve a variety of time series forecasting problems.

## Data availability statement

The original contributions presented in this study are included in this article/supplementary material, further inquiries can be directed to the corresponding authors.

## Author contributions

HL: methodology and writing—original draft. GZ: writing—review and editing. YZ: analysis of experimental results and software. HH: review and editing. XW: testing of experimental results. All authors contributed to the article and approved the submitted version.

## Footnotes

1
http://shumo.neepu.edu.cn
